# A Dominant Negative OsKAT2 Mutant Delays Light-Induced Stomatal Opening and Improves Drought Tolerance without Yield Penalty in Rice

**DOI:** 10.3389/fpls.2017.00772

**Published:** 2017-05-12

**Authors:** Seok-Jun Moon, Hyun Y. Kim, Hyunsik Hwang, Jin-Ae Kim, Yongsang Lee, Myung K. Min, In S. Yoon, Taek-Ryoun Kwon, Beom-Gi Kim

**Affiliations:** Gene Engineering Division, National Institute of Agricultural Sciences, Rural Development AdministrationJeonju, South Korea

**Keywords:** OsKAT2, rice, inward rectifying K channel, guard cell, stomatal behavior, dominant negative mutant

## Abstract

Stomata are the main gateways for water and air transport between leaves and the environment. Inward-rectifying potassium channels regulate photo-induced stomatal opening. Rice contains three inward rectifying shaker-like potassium channel proteins, OsKAT1, OsKAT2, and OsKAT3. Among these, only *OsKAT2* is specifically expressed in guard cells. Here, we investigated the functions of OsKAT2 in stomatal regulation using three dominant negative mutant proteins, OsKAT2(T235R), OsKAT2(T285A) and OsKAT2(T285D), which are altered in amino acids in the channel pore and at a phosphorylation site. Yeast complementation and patch clamp assays showed that all three mutant proteins lost channel activity. However, among plants overexpressing these mutant proteins, only plants overexpressing OsKAT2(T235R) showed significantly less water loss than the control. Moreover, overexpression of this mutant protein led to delayed photo-induced stomatal opening and increased drought tolerance. Our results indicate that OsKAT2 is an inward- rectifying shaker-like potassium channel that mainly functions in stomatal opening. Interestingly, overexpression of OsKAT2(T235R) did not cause serious defects in growth or yield in rice, suggesting that OsKAT2 is a potential target for engineering plants with improved drought tolerance without yield penalty.

## Introduction

Cationic nutrients such as K^+^, Ca^2+^, and Mg^2+^ are essential for physiological processes such as plant growth and cellular homeostasis (Dreyer and Uozumi, 2011). Among these, K^+^ is a crucial plant nutrient and the most abundant cation in plants. K^+^ functions as an osmoticum that contributes to cellular turgor adjustment and the control of stomatal opening through the selective movement and redistribution of this cation (Ashley et al., 2006; Szczerba et al., 2009; Dreyer and Uozumi, 2011). Numerous K^+^ transporter systems related to the uptake and release of K^+^ from the cell have been reported in various plant species (Baizabal-Aguirre et al., 1999; Szczerba et al., 2009). Shaker-like K^+^ channels function in stomatal opening and closing through K^+^ uptake and export in guard cells (Maser et al., 2001; Kim et al., 2015). To date, 9 and 11 genes encoding shaker-like potassium channels have been identified in Arabidopsis and rice, respectively (Amrutha et al., 2007). The first shaker-like potassium channel genes, Arabidopsis *AtKAT1* and *AtAKT1*, were isolated and investigated by functional complementation of yeast mutants defective in potassium uptake. AtKAT1, a typical inward-rectifying shaker-like potassium channel, is predominantly expressed in guard cells and plays a role in potassium uptake in guard cells when stomata open (Anderson et al., 1992; Schachtman et al., 1992; Sentenac et al., 1992). Rice contains three inward rectifying shaker-like K^+^ channel proteins (OsKAT1, OsKT2, and OsKAT3). OsKAT1 increases salt tolerance in a salt-sensitive yeast mutant and maintains ion homeostasis during salt stress in cultured rice cells (Obata et al., 2007). OsKAT2 is specifically expressed in rice guard cells, where it might function as an inward rectifying shaker-like potassium channel and might be a functional ortholog of Arabidopsis AtKAT1 (Hwang et al., 2013). However, the role of OsKAT2 in stomatal opening has not yet been verified genetically.

Plant shaker-like K^+^ channels possess a conserved sequence motif (TXXTXGYG), a hallmark for a critical function in channel selectivity in both plant and animal cells (Ward et al., 2009). The overexpression of AtKAT1 with two single point mutations (T256R) and G262K) in the GYGD motif and surrounding amino acids of this motif region suppressed time-dependent inward currents in *Xenopus oocytes* (Baizabal-Aguirre et al., 1999). Overexpressing these AtKAT1 mutant proteins in Arabidopsis depressed light-induced stomatal opening and reduced K^+^ uptake, and therefore, these dominant negative mutant lines exhibited less water loss than the control (Kwak et al., 2001). In addition, a single point mutation of the threonine at position 306 in AtKAT1, which is a target site for ABA-activated SnRK2.6 protein kinase, reduced the K^+^ transport uptake activity of AtKAT1 in *Xenopus oocyte* and yeast systems (Sato et al., 2009, 2010). Thus, these amino acid sites are good candidates for constructing dominant negative mutants to help identify the function of *OsKAT2* in rice guard cells.

Water deficiency is a major factor limiting rice yields, as rice production depends on the use of large amounts of water (Kim et al., 2012). Stomata are the main gateways through which air and water evaporate into the atmosphere. Accordingly, Schroeder et al. (2001) proposed that engineering guard cells could improve drought tolerance in plants. If OsKAT2 is a functional inward rectifying potassium channel that is active mainly in rice guard cells, it would be a good candidate for guard cell engineering to improve drought tolerance and water use efficiency.

Here, we investigated the function of OsKAT2 in stomatal regulation in rice using dominant negative mutant proteins in an *in planta* system and found that OsKAT2 functions in photo-induced stomatal opening. We also found that plants overexpressing dominant negative OsKAT2 mutant proteins exhibited reduced water loss in detached leaves and enhanced drought tolerance without yield penalty. Thus, *OsKAT2* is a good target gene for engineering to improve drought tolerance in rice.

## Materials and Methods

### Plant Materials and Growth Conditions

Rice (*Oryza sativa* L. ssp. *japonica* cv. Dongjin) was used for *Agrobacterium*-mediated rice transformation in this study. Transgenic rice plants were grown on soil in the greenhouse and on MS (Murashige and Skoog) agar [per liter: 4.4 g MS salt, 30 g sucrose, 0.5 g 2-(*N*-Morpholino)ethanesulfonic acid (MES), 8 g plant agar, pH 5.8] in a growth chamber maintained at 25°C and 60% relative humidity under long-day conditions (under a 16 h light/8 h dark cycle).

### Yeast Complementation Assay

Wild-type Os*KAT2*, Os*KAT2* T235R, Os*KAT2* T285A, and Os*KAT2* T285D full-length cDNAs were introduced into the pYES52-DEST yeast expression vector (Invitrogen, USA) and transformed into K^+^ uptake-deficient yeast strain CY162 (*MATa ade2 ura3 leu2 his3 his4 trk1 delta trk2 delta::pCK64*) (Anderson et al., 1992). Yeast transformation was performed using LiCl as described by Chen et al. (1992). Transformed yeast cells were selected on minimal solid medium (SDG medium, i.e., minimal medium supplemented with amino acids except uracil plus 2% galactose, 1% raffinose, and 2% agarose). Complementation tests were performed by spotting serially diluted 5 μl cell suspensions (A_600_ = 0.2) on solid medium containing different concentrations of KCl, followed by incubation at 28°C for 2–3 days.

### Heterologous Expression of OsKAT2 in HEK293 Cells

HEK293 cells were cultured in Dulbecco’s modified Eagle’s medium (DMEM with 4,500 mg/l glucose; Gibco, USA) containing 2 mM glutamine, 100 U/ml penicillin/streptomycin and 10% FBS (Invitrogen, USA). The cells were transfected with 6 μg of mammalian expression vector pcDNA6.2 V5-DEST (Invitrogen, USA) containing wild-type Os*KAT2*, Os*KAT2* T235R, Os*KAT2* T285A, and Os*KAT2* T285D according to the manufacturer’s protocol using Lipofectamine 2000 (Invitrogen, USA). Transfected cells were incubated in DMEM medium and maintained at 37°C in a humidified incubator in the presence of 5% CO_2_ (Sanyo, Japan). For electrophysiological studies, cells were spread on cover slips coated with 0.1% poly-D-lysine (Sigma-Aldrich, USA). The experiments were performed using cells incubated for 1–2 days after transfection.

### Patch Clamp Measurements

Whole-cell voltage clamp recording was performed at room temperature to measure K^+^-uptake capacity. Inward currents were measured using an EPC-8 amplifier and Patch master software (Both from HEKA Instruments, Germany). The patch pipettes (World Precision Instruments, Inc.) were constructed using a PP-830 puller (Narishige, Tokyo). When filled with pipette solution, the resistance of the pipettes was 3–6 MΩ (always less than 10 MΩ). The pipette capacitance was compensated after the formation of a giga seal. The recording chamber was continuously superfused (1–2 ml/min). Data were low-pass filtered at 2 kHz and acquired using the Patch master program. The standard bath solution contained 170 mM K-gluconate, 1 mM CaCl_2_, 2.5 mM MgCl_2_, and 10 mM HEPES/Tris, pH 6.8. The pipette solution contained 150 mM K-gluconate, 20 mM KCl, 1 mM CaCl_2_, 10 mM HEPES, 10 mM EGTA and 4 mM Mg-ATP/Tris, pH 7.4. The protocol for recording potassium channel currents consisted of stepping the membrane potential from a holding potential of -20 mV to the test potential (ranging from -140 to +40 mV) for 1 s in 20 mV increments at 2 s intervals. To minimize changes in offset potentials during bath solution exchanges, 3 M-KCl agar salt bridges were used for the reference electrode. Data are presented as means ± standard error (SEM).

### Generation of Dominant Negative OsKAT2 Mutant Lines and Phenotype Observations

Genes encoding OsKAT2 mutant proteins with a point mutation at the 235th amino acid (T235R) and 285th amino acid (T285A and T285D) were cloned into pGA2897, a plant expression vector, driven by the maize *UBIQUITIN* promoter (Kim et al., 2012) (Supplementary Figure S4). Constructs harboring these *OsKAT2* mutants were transferred into *Agrobacterium tumefaciens* LBA4404 by electroporation using a MicroPulser Eletroporator (BioRad, USA). The *Agrobacterium* was transformed into rice as previously reported (Han et al., 2012). T0 transgenic rice plants were selected on hygromycin medium. Among transgenic rice lines, transgenic plants overexpressing the *OsKAT2* mutant genes were chosen for further study via quantitative RT-PCR (Kim et al., 2015).

For the drought tolerance assay, 25 dominant negative mutant plants and 25 wild-type plants were grown together in the same pots (L165 × W80 × H70 mm) filled with soil for 2 weeks in the greenhouse. Watering was then withheld from the plants for 7 days, followed by re-watering. Drought tolerance was assessed by determining the survival rates during the recovery period after 14 days of re-watering.

### Detached Leaf Water Loss Assay

To perform the water loss assay using detached leaves, transgenic and wild-type plants were incubated in a growth chamber for 10 days. The third leaves were detached from the plants and dried in weighing dishes under white light in a tissue culture room (26 ± 2°C). The fresh weights of the leaves were measured at specific time intervals. Water loss was calculated as the percentage of initial fresh weight at each time point. Each experiment was repeated twice.

### Evaluation of Agronomic Characteristics

To evaluate agronomic characteristics such as plant height, culm length, number of panicles and total seed weight, six independent T2 (in 2014 year) and T3 (in 2015 year) lines of transgenic plants overexpressing OsKAT2(T235R) were grown in a rice paddy field as described previously (Kim et al., 2014). At 1 month after sowing, transgenic and wild-type seedlings were transplanted at a distance of 20 cm, with a single seedling per hill, in a rice paddy field. When the rice seeds had ripened, the agronomic characteristics were measured in 20 plants per each transgenic line.

### Measurement of Stomatal Conductance

Leaf stomatal conductance was measured using a portable photosynthesis system (LI-6400, LI-Cor, Inc., Lincoln, NE, USA). OsKAT2(T235R) mutant lines were grown in soil for 45 days and stomatal conductance was measured using one of three fully expanded leaves at the top of the main stem, which was selected at random(Kusumi et al., 2012). The chamber conditions were maintained at 28°C, relative humidity of 40–60% and CO_2_ concentration of 400 ppm using the program provided with the LI-6400 system. Light intensity was set at 1000 μmol/m^2^⋅s in the chamber.

## Results

### OsKAT2 with a Single Amino Acid Mutation at Amino Acid 235 or 285 Cannot Complement a K^+^ Uptake-Deficient Yeast Mutant

**FIGURE 1:**
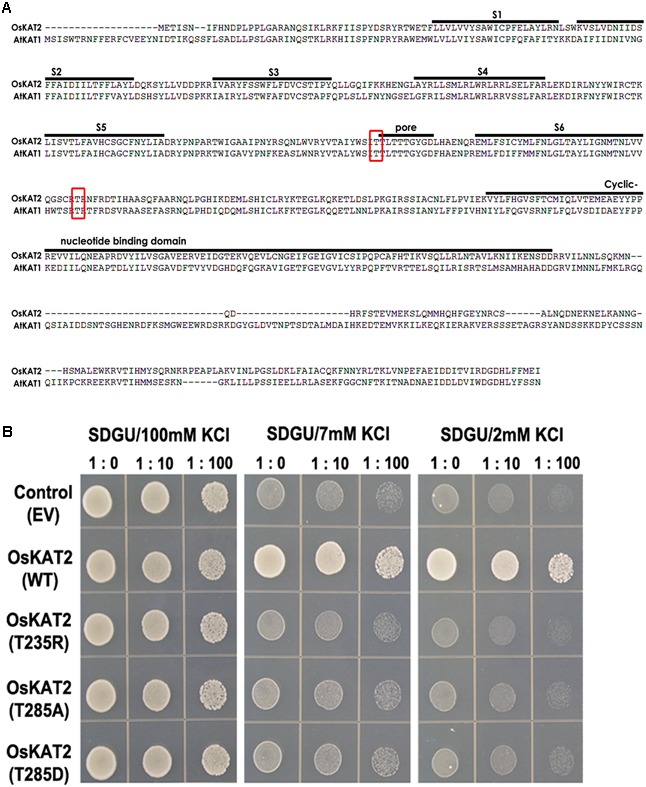
**Amino acid sequence comparisons of OsKAT2 and AtKAT1 proteins, and complementation analysis of OsKAT2 mutants using potassium uptake-deficient yeast. (A)** The consensus TxxTxGYG motif in the K^+^-selective pore-forming loop region is labeled “pore.” The regions indicated by solid lines are putative transmembrane domains (S1–S6). The red box represents the positions of the single amino acid mutations in OsKAT2. **(B)** The CY162 yeast strain was transformed with empty vector (EV, pYES-DEST52), OsKAT2 (wild-type) and OsKAT2 single amino-acid mutant (T235R, T285D, and T285A). Ten-fold serial dilutions of yeast cell suspensions were spotted onto solid SDGU medium containing 2, 7, or 100 mM KCl. Accession numbers are follows: OsKAT2, XP015614100; AtKAT1, NP_199436.

To identify the function of OsKAT2, we constructed dominant-negative OsKAT2 mutant proteins (Supplementary Figure S3). First, we compared the amino acid sequences of OsKAT2 versus AtKAT1 and found that the 235th and 285th threonine of OsKAT2 corresponded to the 256th and 306th threonine of AtKAT1 (**Figure 1A**). We constructed three proteins with single amino-acid mutations at these two threonine sites, including threonine substituted with arginine (T235R), alanine (T285A) and aspartic acid (T285D), and performed a complementation assay using K^+^ uptake-deficient yeast strain, CY162, which was previously reported to be complemented by OsKAT2 (Hwang et al., 2013). When CY162 yeast cells were transformed with empty vector (pYES52-DEST), the K^+^ uptake deficiency of the cells was not complemented, but in yeast cells expressing wild-type OsKAT2, the K^+^ uptake deficiency was complemented, as the cells grew on medium containing 2 mM K^+^, as previously reported (**Figure 1B**). However, the three mutant proteins, T235R, T285A and T285D, failed to complement the CY162 yeast mutant on medium containing 2 mM K^+^ (**Figure 1B**). These results suggest that these mutant proteins might have lost their K^+^ channel activity.

### OsKAT2 Mutant Proteins Lack Voltage-Dependent K^+^ Channel Activity

To further confirm that the OsKAT2 mutant proteins had lost their channel activity, we measured the voltage-dependent potassium channel activity of HEK293 cells transiently expressing the OsKAT2 mutant proteins using a whole-cell patch clamp experiment. To examine the K^+^ uptake activity of the three OsKAT2 mutant channels, we measured the K^+^ currents in HEK293 cells expressing each K^+^ channels.

**FIGURE 2:**
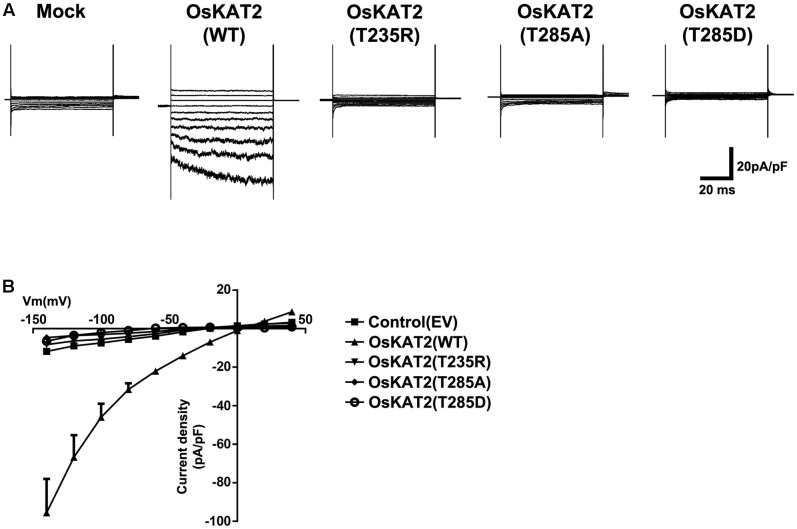
**Patch-clamp analysis to measure channel activity in the OsKAT2 single amino acid mutants. (A)** Representative current traces show the channel activity of each OsKAT2 protein. HEK293 cells with empty vector (EV, pcDNA6.2-EmGFP) or expression constructs for wild-type OsKAT2, OsKAT2(T235R), OsKAT2(T285A), or OsKAT2(T285D) were recorded by whole-cell patch-clamp analysis. Currents were obtained by changing the membrane voltage from a holding potential of –20 mV following test pulses from –140 to + 60 mV in 20 mV increments. **(B)** Steady-state *I–V* relations of current density collected at the end of the test pulse (lower panel). Results are means ± standard error (SEM).

In control experiments, when empty vector (EV, pcDNA6.2-EmGFP)-transfected HEK293 cells were examined, an inward current was not induced (**Figure 2A**). By contrast, when wild-type OsKAT2 channels were expressed in the cells, inward currents were significantly induced (**Figure 2A**). Voltage pulses negative to 0 mV elicited slowly activating, voltage-dependent inwardly rectifying currents in Os*KAT2*-expressing cells. However, HEK293 cells expressing each OsKAT2 mutant proteins, i.e., *OsKAT2(T235R)*, *OsKAT2(T285A)* or *OsKAT2(T285D)*, evoked few or no inward currents (**Figure 2A**). To compare the channel activities, we calculated the current-voltage (*I/V*) relationships of steady-state currents. OsKAT2 showed strong inward current density at -140 mV (-95.62 ± 17.59 pA/pF) compared with empty vector (-11.82 ± 0.17 pA/pF). At -140 mV, the current density of OsKAT2 T235R, T285A, and T285D was -8.17 ± 0.17 pA/pF, -4.63 ± 10.92 pA/pF, and -6.56 ± 0.16 pA/pF, respectively (**Figure 2B**). Taken together, these results indicate that the *T235R*, *T285A*, and *T285D* mutants of OsKAT2 have lost their functions as inwardly rectifying potassium channels.

### Overexpression of an OsKAT2 Mutant Protein Reduces Water Loss in Transgenic Rice

**FIGURE 3:**
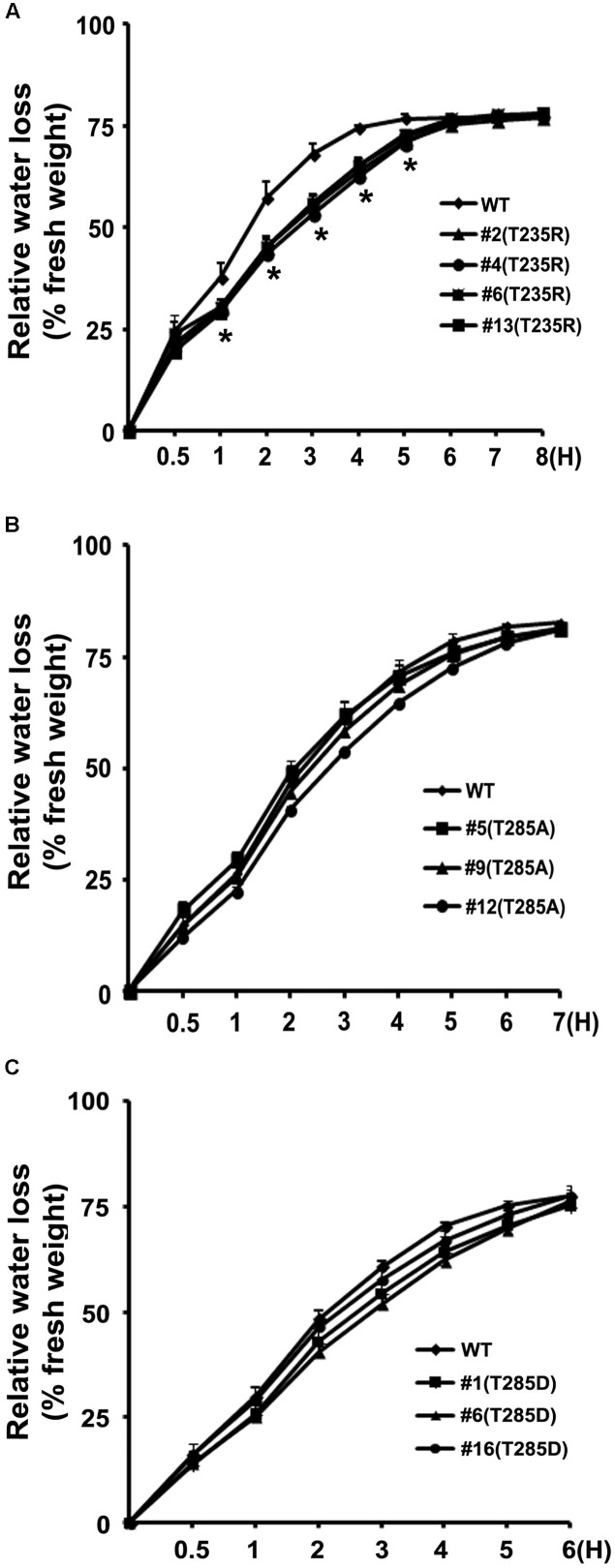
**Water loss assay of transgenic rice plants overexpressing OsKAT2 mutant proteins. (A)** Transgenic rice plants overexpressing OsKAT2(T235R), **(B)** transgenic rice plants overexpressing OsKAT2(T285A), **(C)** transgenic rice plants overexpressing OsKAT2(T285D). Relative water loss rates in detached leaves from wild-type and T2 transgenic rice plants expressing dominant-negative OsKAT2 mutant proteins under drought-stress conditions. Water loss is indicated as the percentage of weight loss versus the initial fresh weight. Results are means ± standard error (SEM). Asterisks indicate a significant difference (one-way ANOVA with Turkey’s test, ^∗^*p*-value < 0.05).

To investigate whether OsKAT2 functions in rice stomatal regulation in rice, we generated transgenic rice plants overexpressing the dominant negative OsKAT2 mutant proteins (T235R, T285A, and T285D) under the control of the maize *UBIQUITIN* promoter. We examined the levels of mutant *OsKAT2* expression in transgenic rice plants overexpressing the three OsKAT2 mutant proteins individually via qRT-PCR analysis (Supplementary Figures S1, S2). We then selected three or four independent transgenic rice plants for leaf water loss analysis (**Figure 3**). Leaf water loss rates were calculated by measuring the fresh weights of detached leaves every hour over the course of 8 h. As shown **Figure 3A**, during the first 5 h of measurements, detached leaves of the dominant negative OsKAT2(T235R) mutant-overexpressing transgenic rice lost less water than non-transformed control (‘Dongjin’). After 3 h of treatment, the fresh weights of control leaves decreased by approximately 68%, but the leaves of the three dominant negative OsKAT2(T235R) mutant-overexpressing transgenic rice lines lost only approximately 53–55% of their fresh weights (**Figure 3A**). By contrast, detached leaves of plants overexpressing the two other dominant negative OsKAT2(T285A and T285D) mutant proteins showed similar water loss rates to those of non-transformed control plants (**Figures 3B,C**). These results suggest that expressing dominant negative OsKAT2(T235R) may cause defects in stomatal regulation, but expressing the two other mutant proteins (T285A and T285D) does not seriously affect stomatal regulation in rice.

### Overexpression of OsKAT2(T235R) Delays Light-Dependent Stomatal Opening

**FIGURE 4:**
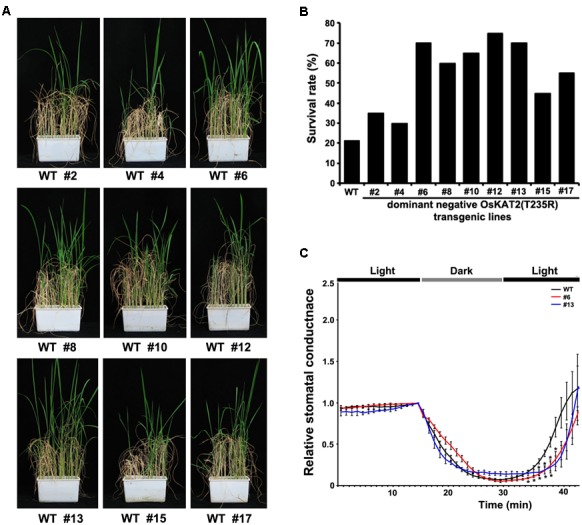
**Drought tolerance and stomatal conductance analyses of transgenic rice overexpressing OsKAT2(T235R). (A)** Drought tolerance assays were performed with 1-month-old T2 transgenic rice plants expressing dominant-negative OsKAT2(T235R). Watering was stopped for 7 days and resumed for 2 weeks. **(B)** Survival rates of wild-type and transgenic rice plants expressing dominant-negative OsKAT2(T235R) under drought-stress conditions. **(C)** Time course of stomatal responses in wild-type and transgenic rice plants expressing OsKAT2(T235R) under light and dark conditions. Stomatal conductance was monitored in 45-day-old rice leaves that were fully expanded at the top of the main stem using the LI-6400 portable photosynthesis system. Asterisks indicate a significant difference (one-way ANOVA with Turkey’s test, ^∗^*p*-value < 0.05).

Since dominant negative OsKAT2(T235R) mutant rice showed lower water loss rates than the control, we measured changes in stomatal conductance in these plants. Under constant dark or light conditions, little difference in stomatal conductance was detected between wild-type and mutant plants. However, when the plants were transferred from darkness to light, the stomatal conductance pattern was quite different between wild-type and the mutant (**Figure 4C**). Wild-type plants began to show an increase of stomatal conductance at 35 min after transfer from darkness to light, whereas stomatal conductance did not increase in mutant plants until after around 40 min. Taken together, these results indicate that OsKAT2(T235R)-overexpressing rice showed delayed light-induced stomatal opening.

### Performance of OsKAT2(T235R)-Overexpressing Transgenic Rice under Drought Stress

Since plants overexpressing the dominant negative OsKAT2(T235R) mutant protein exhibited reduced water loss and delayed light-dependent stomatal opening, we assayed the drought tolerance phenotypes of these plants. For drought stress treatment, we withheld watering from 2-week-old transgenic and wild-type rice plants for 7 days and resumed watering for 14 days in the greenhouse. Fourteen days after re-watering, the OsKAT2(T235R)-overexpressing transgenic plants showed stronger growth recovery than the control (**Figure 4A**). However, most wild-type rice plants did not survive drought stress/re-watering conditions (**Figure 4A**). We investigated the survival rates of transgenic and wild-type rice plants. Except for transgenic lines #2, #4 and #15, more than 50% of the OsKAT2(T235R)-overexpressing transgenic plants survived drought-stress treatment compared with 20% of wild-type plants (**Figure 4B**). Therefore, the dominant negative OsKAT2(T235R) mutant-overexpressing transgenic rice plants had improved drought tolerance compared to wild-type plants.

### Agronomic Performance of Transgenic Rice Plants Overexpressing Dominant Negative OsKAT2(T235R) Mutant

**FIGURE 5:**
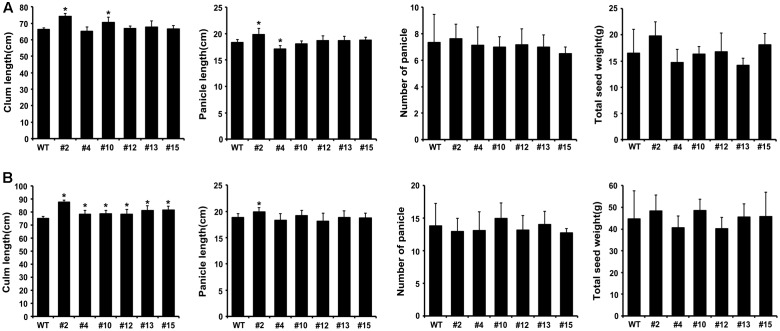
**Growth characteristics of transgenic rice plants expressing dominant-negative OsKAT2(T235R) mutant protein.** Culm length panicle length, number of panicles and total seed weight of wild-type and T2 **(A)**, T3 **(B**) transgenic rice plants were measured in approximately 16-week-old plants grown in a paddy field under normal conditions. Asterisks indicate a significant difference (one-way ANOVA with Turkey’s test, ^∗^*p*-value < 0.05).

To investigate whether the expression of mutant OsKAT2(T235R) affects growth and yield production in rice, we grew six independent T2 and T3 transgenic rice plants under normal field conditions for 2 years. As shown in **Figure 5**, the expression of OsKAT2(T235R) did not affect agronomic traits such as culm length, number of panicles or panicle length, as the OsKAT2(T235R)-overexpressing transgenic rice plants showed quite similar agronomic phenotypes to those of wild-type (**Figures 5A,B**).

To compare total seed weight per plant, we harvested and weighed seeds from transgenic and wild-type rice plants. Except for two transgenic lines (#4 and #12), the total seed weights of the OsKAT2(T235R)-overexpressing plants (#2, #10, #13, #15) were similar or little higher (45.5 to 48.4 g) than those of wild-type (44.8 g) plants (**Figure 5B**). These results indicate that expressing the dominant-negative OsKAT2(T235R) mutant protein in rice does not markedly alter plant growth and development.

## Discussion

Shaker-type channels in plants can be subdivided into three major subgroups based on channel characteristics: (1) inward rectifiers, which mediate K^+^ uptake; (2) weak rectifiers, which have specific gating properties and (3) outward rectifiers (Czempinski et al., 1999; Zimmermann and Sentenac, 1999; Gambale and Uozumi, 2006; Ward et al., 2009). Among these, some inward rectifier K^+^ channels function in stomatal opening (Dietrich et al., 2001; Very and Sentenac, 2003). Although AtKAT1 was shown to play a role in light-dependent stomatal opening in Arabidopsis, the K^+^ channel that functions in light-dependent stomatal opening in rice was previously unknown (Kwak et al., 2001). *OsKAT2* is specifically expressed in rice guard cells and shows typical inward-rectifying K^+^ channel activity, like that of Arabidopsis *AtKAT1* (Hwang et al., 2013). Thus, we investigated the function of OsKAT2 genetically using a dominant negative mutant of OsKAT2.

### 235th and 285th Threonine of OsKAT1 are Crucial Amino Acids to Regulate Channel Activity

We identified mutation target amino acids, T235 and T285 of OsKAT2, corresponding T256 and T306 of AtKAT1, through sequence comparisons between AtKAT1 and OsKAT2. The T235 is located in the K^+^ channel pore region between S5 and S6 domain, whereas the T285 is located in C-terminal region protruding to cytosolic region (**Figure 1A**). T235 is mutated into R with a positively charged side chain and T285 is mutated into A and D which leads to be the removal of the phosphorylation site and the addition of phosphomimetic, respectively (Supplementary Figure S3). All three mutant proteins have lost their channel activity completely. In yeast, overexpression of three mutant genes cannot complement the K^+^ uptake deficient mutant compared to wild-type OsKAT1. Also, overexpression of these three mutants does not show any inward current in HEK293 cell. Thus, we can conclude that T235 might be a crucial amino acid in forming the gate of a potassium channel, OsKAT1 and T285 might play a role in channel activity regulation through signaling transduction of a potassium channel, OsKAT1.

### Overexpression of OsKAT2(T235R) Mutant Gene Causes the Defects of Stomatal Regulation in Rice

We expected that plants overexpressing these mutant proteins which lost channel activity would have defects in stomatal regulation and would show reduced water loss phenotype. However, transgenic plants overexpressing OsKAT2(T285A) and OsKAT2(T285D) did not show serious differences in water loss compared to wild-type meaning that they might not have defects in stomatal regulation. However, OsKAT2(T235R)-overexpressing plants showed significantly reduced water loss phenotype. It is possible that OsKAT2(T285A) and OsKAT2(T285D) mutant proteins exist as homo-tetramers due to the lack of endogenous wild-type OsKAT2 in heterologous systems such as yeast and HEK293 cells. Despite lacking channel activity, these mutant proteins might not induce serious phenotypes in plants if they can form hetero-tetramers with endogenous wild-type OsKAT2, which could complement the effects of the mutants. By contrast, the OsKAT2(T235R) mutant proteins might disrupt K^+^ channel activity by forming hetero-tetramers with wild-type OsKAT2, but preventing the formation of intact pores *in planta*.

The defects of stomata opening in rice overexpressing OsKAT2(T235R) were proved directly by measuring stomata conductance. We could not see any big difference of stomata conductance in constant dark or light condition. However, there is the quite significant difference of stomata conductance under the condition which transits from dark to light. In Arabidopsis, the expression of a dominant negative AtKAT1 mutant protein with T256R amino acid substitutions reduced light-induced stomatal opening in guard cells and resulted in a decline in water loss from the leaves (Baizabal-Aguirre et al., 1999; Kwak et al., 2001). Thus, plants overexpressing OsKTA2(T235R) mutant protein might be delayed in stomatal opening both in light induced condition and in water deficient condition.

### Overexpression of OsKAT2(T235R) Mutant Gene Improves the Drought Tolerance without Yield Penalty in Rice

The dominant negative mutant of OsKAT2(T235R) delay the stomatal opening in several conditions. Thus, the dominant negative mutants showed improved drought tolerance even though there is no significant correlation between gene expression level and drought tolerance (**Figure 4** and Supplementary Figure S2). Delayed guard cell opening can improve the drought tolerance but can cause the defects in CO_2_ uptake and photosynthesis. Also, if dominant negative mutant of OsKAT1 functions in different tissues such as root, it will cause serious defects in plant growth. However, unexpectedly agronomic traits of dominant negative mutant OsKAT2(T235R) was not much different from control in growth, development and grain yields (**Figure 5**). This result suggest that OsKAT2 might function specifically in guard cell.

Our results suggest that the threonine at position 235 in OsKAT2 plays an important role in K^+^ channel pore formation and that OsKAT2 is a potassium channel that regulates light-induced stomatal opening in rice. We found that dominant negative mutant of OsKAT2(T234R) is a good target to improve drought tolerance in rice via guard cell engineering without yield penalty.

## Author Contributions

S-JM managed the all materials and measured agronomic traits and drought tolerance, HK performed the patch clamp works, HH carried out the yeast works and J-AK and MM did the water loss assay and YL measured the stomata conductance. IY and T-RK revised manuscript and designed project and S-JM, HK, B-GK wrote the manuscript, analyzed data and designed experiments. All authors read and approved the manuscript.

## Conflict of Interest Statement

The authors declare that the research was conducted in the absence of any commercial or financial relationships that could be construed as a potential conflict of interest.

## References

[B1] Amrutha R. N. Sekhar P. N. Varshney R. K. Kishor P. B. K. (2007). Genome-wide analysis and identification of genes related to potassium transporter families in rice (*Oryza sativa* L.). *Plant Sci.* 172 708–721. 10.1016/j.plantsci.2006.11.019

[B2] Anderson J. A. Huprikar S. S. Kochian L. V. Lucas W. J. Gaber R. F. (1992). Functional expression of a probable *Arabidopsis thaliana* potassium channel in *Saccharomyces cerevisiae*. *Proc. Natl. Acad. Sci. U.S.A.* 89 3736–3740. 10.1073/pnas.89.9.3736 1570292 PMC525565

[B3] Ashley M. K. Grant M. Grabov A. (2006). Plant responses to potassium deficiencies: a role for potassium transport proteins. *J. Exp. Bot.* 57 425–436. 10.1093/jxb/erj034 16364949

[B4] Baizabal-Aguirre V. M. Clemens S. Uozumi N. Schroeder J. I. (1999). Suppression of inward-rectifying K+ channels KAT1 and AKT2 by dominant negative point mutations in the KAT1 alpha-subunit. *J. Membr. Biol.* 167 119–125. 10.1007/s002329900476 9916143

[B5] Chen D. C. Yang B. C. Kuo T. T. (1992). One-step transformation of yeast in stationary phase. *Curr. Genet.* 21 83–84. 10.1007/BF00318659 1735128

[B6] Czempinski K. Gaedeke N. Zimmermann S. Muller-Rober B. (1999). Molecular mechanisms and regulation of plant ion channels. *J. Exp. Bot.* 50 955–966. 10.1093/jxb/50.Special_Issue.955

[B7] Dietrich P. Sanders D. Hedrich R. (2001). The role of ion channels in light-dependent stomatal opening. *J. Exp. Bot.* 52 1959–1967. 10.1093/jexbot/52.363.1959 11559731

[B8] Dreyer I. Uozumi N. (2011). Potassium channels in plant cells. *FEBS J.* 278 4293–4303. 10.1111/j.1742-4658.2011.08371.x 21955642

[B9] Gambale F. Uozumi N. (2006). Properties of shaker-type potassium channels in higher plants. *J. Membr. Biol.* 210 1–19. 10.1007/s00232-006-0856-x 16794778

[B10] Han S. Y. Shin D. J. Moon S. J. Jeon S. A. Byun M. O. Kim B. G. (2012). Optimization of *Agrobacterium*-mediated transformation in japonica-type rice *Oryza Sativa* L. cv. Dongjin for high efficiency. *Korean J. Breed. Sci.* 44 221–228.

[B11] Hwang H. Yoon J. Kim H. Y. Min M. K. Kim J. A. Choi E. H. (2013). Unique features of two potassium channels, OsKAT2 and OsKAT3, expressed in rice guard cells. *PLoS ONE* 8:e72541 10.1371/journal.pone.0072541 PMC3742606 23967316

[B12] Kim H. Hwang H. Hong J. W. Lee Y. N. Ahn I. P. Yoon I. S. (2012). A rice orthologue of the ABA receptor, OsPYL/RCAR5, is a positive regulator of the ABA signal transduction pathway in seed germination and early seedling growth. *J. Exp. Bot.* 63 1013–1024. 10.1093/jxb/err338 22071266

[B13] Kim H. Lee K. Hwang H. Bhatnagar N. Kim D. Y. Yoon I. S. (2014). Overexpression of PYL5 in rice enhances drought tolerance, inhibits growth, and modulates gene expression. *J. Exp. Bot.* 65 453–464. 10.1093/jxb/ert397 24474809 PMC3904710

[B14] Kim H. Y. Choi E. H. Min M. K. Hwang H. Moon S. J. Yoon I. S. (2015). Differential gene expression of two outward-rectifying shaker-Like potassium channels OsSKOR and OsGORK in rice. *J. Plant Biol.* 58 230–235. 10.1007/s12374-015-0070-4

[B15] Kusumi K. Hirotsuka S. Kumamaru T. Iba K. (2012). Increased leaf photosynthesis caused by elevated stomatal conductance in a rice mutant deficient in SLAC1, a guard cell anion channel protein. *J. Exp. Bot.* 63 5635–5644. 10.1093/jxb/ers216 22915747 PMC3444276

[B16] Kwak J. M. Murata Y. Baizabal-Aguirre V. M. Merrill J. Wang M. Kemper A. (2001). Dominant negative guard cell K+ channel mutants reduce inward-rectifying K+ currents and light-induced stomatal opening in arabidopsis. *Plant Physiol.* 127 473–485. 10.1104/pp.010428 11598222 PMC125083

[B17] Maser P. Thomine S. Schroeder J. I. Ward J. M. Hirschi K. Sze H. (2001). Phylogenetic relationships within cation transporter families of Arabidopsis. *Plant Physiol.* 126 1646–1667. 10.1104/pp.126.4.1646 11500563 PMC117164

[B18] Obata T. Kitamoto H. K. Nakamura A. Fukuda A. Tanaka Y. (2007). Rice shaker potassium channel OsKAT1 confers tolerance to salinity stress on yeast and rice cells. *Plant Physiol.* 144 1978–1985. 10.1104/pp.107.101154 17586689 PMC1949902

[B19] Sato A. Gambale F. Dreyer I. Uozumi N. (2010). Modulation of the Arabidopsis KAT1 channel by an activator of protein kinase C in *Xenopus laevis* oocytes. *FEBS J.* 277 2318–2328. 10.1111/j.1742-4658.2010.07647.x 20423459

[B20] Sato A. Sato Y. Fukao Y. Fujiwara M. Umezawa T. Shinozaki K. (2009). Threonine at position 306 of the KAT1 potassium channel is essential for channel activity and is a target site for ABA-activated SnRK2/OST1/SnRK2.6 protein kinase. *Biochem. J.* 424 439–448. 10.1042/BJ20091221 19785574

[B21] Schachtman D. P. Schroeder J. I. Lucas W. J. Anderson J. A. Gaber R. F. (1992). Expression of an inward-rectifying potassium channel by the Arabidopsis KAT1 cDNA. *Science* 258 1654–1658. 10.1126/science.8966547 8966547

[B22] Schroeder J. I. Kwak J. M. Allen G. J. (2001). Guard cell abscisic acid signalling and engineering drought hardiness in plants. *Nature* 410 327–330. 10.1038/35066500 11268200

[B23] Sentenac H. Bonneaud N. Minet M. Lacroute F. Salmon J. M. Gaymard F. (1992). Cloning and expression in yeast of a plant potassium ion transport system. *Science* 256 663–665. 10.1126/science.1585180 1585180

[B24] Szczerba M. W. Britto D. T. Kronzucker H. J. (2009). K+ transport in plants: physiology and molecular biology. *J. Plant Physiol.* 166 447–466. 10.1016/j.jplph.2008.12.009 19217185

[B25] Very A. A. Sentenac H. (2003). Molecular mechanisms and regulation of K+ transport in higher plants. *Annu. Rev. Plant Biol.* 54 575–603. 10.1146/annurev.arplant.54.031902.134831 14503004

[B26] Ward J. M. Maser P. Schroeder J. I. (2009). Plant ion channels: gene families, physiology, and functional genomics analyses. *Annu. Rev. Physiol.* 71 59–82. 10.1146/annurev.physiol.010908.163204 18842100 PMC4790454

[B27] Zimmermann S. Sentenac H. (1999). Plant ion channels: from molecular structures to physiological functions. *Curr. Opin. Plant Biol.* 2 477–482. 10.1016/S1369-5266(99)00020-5 10607654

