# Supplementary material for: A Dominant Negative OsKAT2 Mutant Delays Light-Induced Stomatal Opening and Improves Drought Tolerance without Yield Penalty in Rice
Source: Front Plant Sci. 2017 May 12;8:772. doi: 10.3389/fpls.2017.00772 (PMC5427459; doi:10.3389/fpls.2017.00772)
Supplement: Supplementary file 1 [file Data_Sheet_1.pdf]

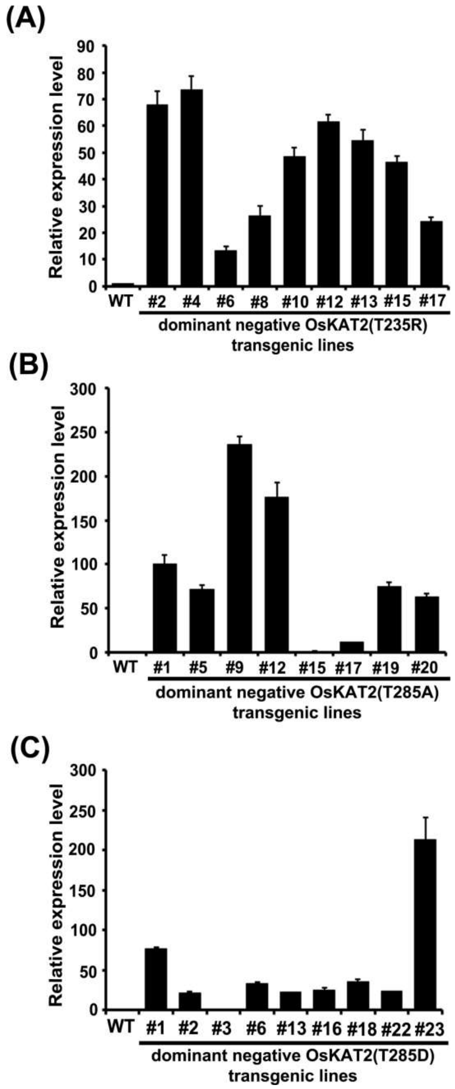

Supplementary Figure S1. Expression analysis of *OsKAT2* in the transgenic rice lines expressing the three dominant-negative OsKAT2 mutant proteins. (A) Expression levels of transgenic rice plants expressing OsKAT2(T235R), (B) Expression level of transgenic rice plants expressing OsKAT2(T285A), (C) Expression level of transgenic rice plants expressing OsKAT2(T285D). Quantitative RT-PCR was performed via the $2^{-\triangle\triangle CT}$ method using the rice *Ubi5* gene as an internal control. The values are the average and SD from three experiments.

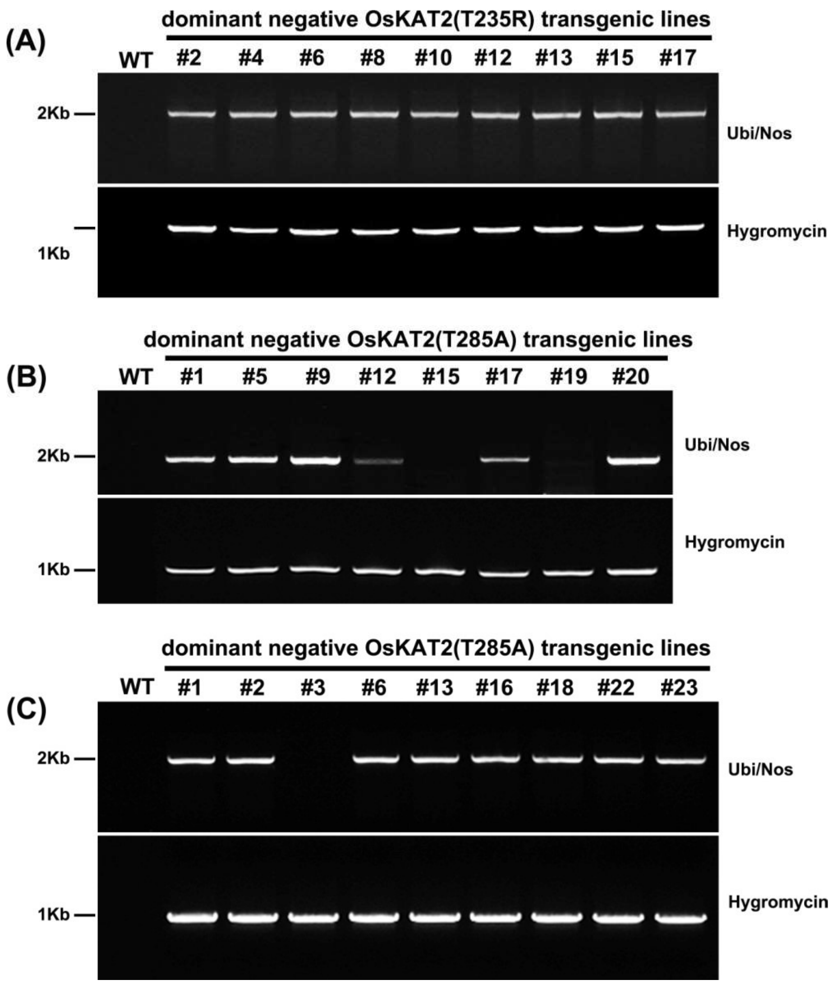

Supplementary Figure S2. Genomic PCR of transgenic rice overexpressing dominant negative mutants. Genomic DNA PCR of WT (Dongjin) and transgenic rice plants overexpressing dominant negative mutants (OsKAT2 T235R, OsKAT2 T285A, OsKAT2 T285D) was performed using Ubi and nos primer set and hygromycin specific primer set. (A) OsKAT2 T235R overexpressing plants, (B) OsKAT2 T285A overexpressing plants, (C) OsKAT2 T285D overexpressing plants.

```
OsKAT(WT)      AGATACCCCAATCCAGCAAGAACATGGATAGGAGCAGCAATACCAAACTACAGATCACAAAA
OsKAT2(T235R)  AGATACCCCAATCCAGCAAGAACATGGATAGGAGCAGCAATACCAAACTACAGATCACAAAA
OsKAT2(T285A)  AGATACCCCAATCCAGCAAGAACATGGATAGGAGCAGCAATACCAAACTACAGATCACAAAA
OsKAT2(T285D)  AGATACCCCAATCCAGCAAGAACATGGATAGGAGCAGCAATACCAAACTACAGATCACAAAA

OsKAT(WT)      TCTGTGGGTTCGATATGTTACGGCAATTTATTGGTCCATAACAACACTCACAACAACTGGTT
OsKAT2(T235R)  TCTGTGGGTTCGATATGTTACGGCAATTTATTGGTCCATACGAACACTCACAACAACTGGTT
OsKAT2(T285A)  TCTGTGGGTTCGATATGTTACGGCAATTTATTGGTCCATAACAACACTCACAACAACTGGTT
OsKAT2(T285D)  TCTGTGGGTTCGATATGTTACGGCAATTTATTGGTCCATAACAACACTCACAACAACTGGTT

OsKAT(WT)      ATGGGGATTTGCATGCAGAGAATCAAAGAGAAATGTTATTTAGCATATGCTACATGCTATTT
OsKAT2(T235R)  ATGGGGATTTGCATGCAGAGAATCAAAGAGAAATGTTATTTAGCATATGCTACATGCTATTT
OsKAT2(T285A)  ATGGGGATTTGCATGCAGAGAATCAAAGAGAAATGTTATTTAGCATATGCTACATGCTATTT
OsKAT2(T285D)  ATGGGGATTTGCATGCAGAGAATCAAAGAGAAATGTTATTTAGCATATGCTACATGCTATTT

OsKAT(WT)      AACCTGGGATTGACAGCATACCTCATAGGTAACATGACAAATCTGGTTGTTCAGGGAAGTTG
OsKAT2(T235R)  AACCTGGGATTGACAGCATACCTCATAGGTAACATGACAAATCTGGTTGTTCAGGGAAGTTG
OsKAT2(T285A)  AACCTGGGATTGACAGCATACCTCATAGGTAACATGACAAATCTGGTTGTTCAGGGAAGTTG
OsKAT2(T285D)  AACCTGGGATTGACAGCATACCTCATAGGTAACATGACAAATCTGGTTGTTCAGGGAAGTTG

OsKAT(WT)      CCGGACCAGGAATTTTAGGGACACAATCCATGCTGCCTCTCAGTTTGCTGCAAGAAATCAGT
OsKAT2(T235R)  CCGGACCAGGAATTTTAGGGACACAATCCATGCTGCCTCTCAGTTTGCTGCAAGAAATCAGT
OsKAT2(T285A)  CCGGGCCAGGAATTTTAGGGACACAATCCATGCTGCCTCTCAGTTTGCTGCAAGAAATCAGT
OsKAT2(T285D)  CCGGGACAGGAATTTTAGGGACACAATCCATGCTGCCTCTCAGTTTGCTGCAAGAAATCAGT

OsKAT(WT)      TGCCTGGACATATTAAGGACGAAATGCTATCTCATATCTGCCTAAGATATAAAACAGAGGGG
OsKAT2(T235R)  TGCCTGGACATATTAAGGACGAAATGCTATCTCATATCTGCCTAAGATATAAAACAGAGGGG
OsKAT2(T285A)  TGCCTGGACATATTAAGGACGAAATGCTATCTCATATCTGCCTAAGATATAAAACAGAGGGG
OsKAT2(T285D)  TGCCTGGACATATTAAGGACGAAATGCTATCTCATATCTGCCTAAGATATAAAACAGAGGGG
```

Supplementary Figure S3. Nucleotide sequence alignment of OsKAT2 mutants after mutation

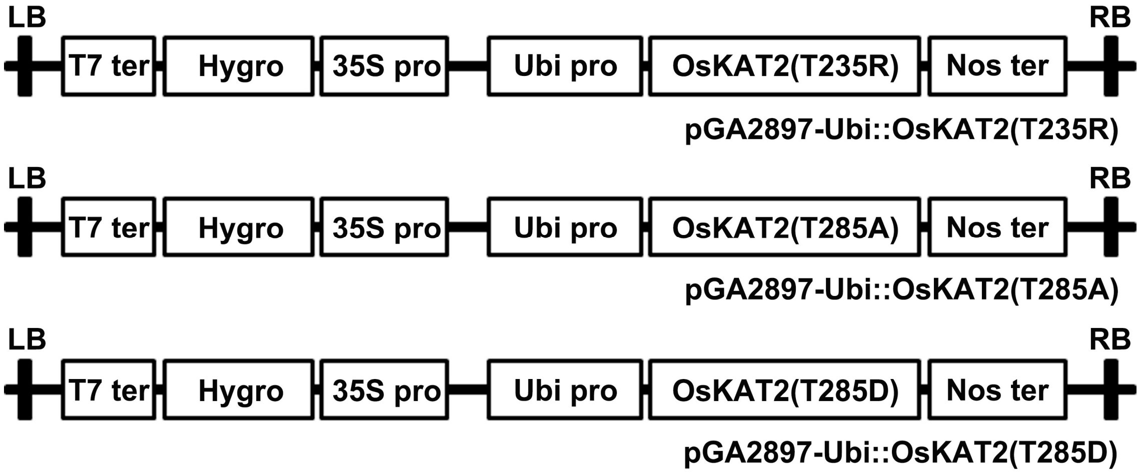

Supplementary Figure S4. Plant expression vector for generating the transgenic rice plants overexpressing the dominant negative OsKAT2 mutants
